# Supplementary material for: Clinical and genetic spectra in patients with dystrophinopathy in Korea: A single-center study
Source: PLoS One. 2021 Jul 23;16(7):e0255011. doi: 10.1371/journal.pone.0255011 (PMC8301650; doi:10.1371/journal.pone.0255011)
Supplement: S1 Table — (PDF) [file pone.0255011.s001.pdf]

S1 Table. Clinical features of 227 patients from 218 unrelated families with dystrophinopathy

ID	Age at the onset	Age at the Dx	Age at the last follow-up	Phenotype	Sex	FH	Scoliosis	Age at the scoliosis surgery	Loss of ambulation	Age at the loss of ambulation	Respiratory discomfort	Age at the start of mechanical ventilation	DCMP	Age at the Dx of DCMP	Pathogenic variant
1	3	8	9	DMD	M	+	+	-	+	9	-	-	-	-	Deletion of exon 45
1-1	3	3	7	UD	M	+	+	-	-	-	-	-	ND	ND	Deletion of exon 45
2	5	14	31	DMD	M	-	+	-	+	7	+	18	+	25	Deletion of exons 46 - 48
3	19	9	19	BMD	M	-	-	-	-	-	-	-	ND	-	c.3803G>A (p.W1268X)
4	9	13	19	BMD	M	-	-	-	+	19	-	-	ND	-	Duplication of exons 3 - 44
5	8	9	17	BMD	M	-	-	-	-	-	-	-	ND	-	Deletion of exons 3 - 7
6	3	3	25	DMD	M	-	+	17	+	10	+	21	+	21	c.829C>T (p.Q277X)
7	7	16	32	DMD	M	-	+	-	+	9	+	20	+	28	c.1652G>A (p.W551X)
8	7	8	25	DMD	M	-	+	-	+	11	+	22	+	23	Deletion of exons 47 - 50
9	4	6	22	DMD	M	+	+	17	+	9	+	18	+	17	Deletion of exon 50
10	5	5	22	DMD	M	+	+	13	+	11	+	20	-	-	Deletion of exons 45 - 50
11	5	25	39	BMD	M	+	-	-	-	-	-	-	ND	ND	Deletion of exons 45 - 47
12	8	19	19	BMD	M	-	+	-	-	-	-	-	-	-	Deletion of exons 45 - 49
13	10	17	17	BMD	M	-	+	-	-	-	-	-	-	-	c.3276+1G>A
14	6	6	21	BMD	M	+	-	-	-	-	-	-	ND	ND	Deletion of exons 45 - 47
14-1	5	4	19	BMD	M	+	-	-	-	-	-	-	ND	ND	Deletion of exons 45 - 47
15	12	19	19	BMD	M	+	-	-	-	-	-	-	-	-	Deletion of exon 49
15-1	5	10	19	BMD	M	+	-	-	-	-	-	-	ND	ND	Deletion of exon 49
16	2	7	8	UD	M	-	+	-	-	-	-	-	ND	ND	c.10108C>T (p.R3370X)
17	5	5	8	DMD	M	-	-	-	+	8	-	-	ND	ND	Deletion of exons 50 – 52
18	3	9	13	DMD	M	-	+	-	+	8	-	-	ND	ND	Deletion of exons 50 - 52
19	4	6	23	DMD	M	+	+	13	+	10	+	14	+	45	c.3728delT (p.L1243fs)
20	7	7	21	DMD	M	-	+	15	+	9	+	16	+	18	Deletion of exons 45 - 50
21	22	26	37	BMD	M	-	-	-	-	-	-	-	-	-	Deletion of exons 45 - 48
22	15	21	31	BMD	M	-	-	-	-	-	-	-	ND	ND	Deletion of exons 45 - 49
23	3	8	8	DMD	M	-	+	-	+	10	-	-	-	-	Duplication of exons 8 - 9
24	7	8	18	DMD	M	-	+	-	+	12	-	-	-	-	c.9563+1G>A
25	7	13	18	BMD	M	+	-	-	-	-	-	-	-	-	Deletion of exons 45 - 47
26	6	8	17	DMD	M	-	+	14	+	10	-	-	+	14	Deletion of exons 46 - 47
27	4	4	17	DMD	M	+	+	16	+	11	-	-	-	-	c.5899C>T (p.R1967X)
28	9	10	11	DMD	M	-	+	-	+	11	-	-	-	-	Deletion of exons 48 - 52
29	6	11	23	BMD	M	-	+	-	+	17	-	-	-	-	c.2169-1G>T
30	2	7	14	DMD	M	-	+	-	+	13	-	-	ND	ND	Deletion of exons 56 - 67
31	20	29	37	BMD	M	+	-	-	-	-	-	-	ND	ND	Deletion of exons 45 - 47
32	5	9	9	DMD	M	+	+	-	+	9	-	-	-	-	Deletion of exons 49 - 54
33	7	6	16	DMD	M	-	+	-	+	10	-	-	+	15	Deletion of promoter region-exon 1
34	19	20	20	BMD	M	-	-	-	-	-	-	-	ND	ND	c.3603+2_3603+3insTA
35	2	6	15	DMD	M	-	+	-	+	8	+	13	+	15	c.9204_9207delCAAA (p.N3068fs)
36	4	7	14	DMD	M	-	-	-	+	8	+	7	-	-	c.6357G>A (p.W2119X)

37	40	43	48	BMD	M	-	-	-	-	-	-	-	ND	ND	c.1704+1G>T
38	2	17	27	DMD	M	-	+	-	+	9	+	18	+	18	c.3277-2A>T
39	18	21	25	BMD	M	-	-	-	-	-	-	-	ND	ND	Deletion of exons 45 - 48
40	4	20	21	DMD	M	-	+	-	+	9	-	-	-	-	c.8111G>A (p.W2704X)
41	13	47	49	BMD	M	-	+	-	+	23	+	48	+	47	c.94-2A>T
42	5	12	23	BMD	M	-	-	-	-	-	-	-	-	-	c.5773G>T (p.E1925X)
43	15	26	50	BMD	M	-	-	-	-	-	-	-	-	-	Deletion of exons 45 - 48
44	26	36	36	Symptomatic carrier	F	-	-	-	-	-	-	-	-	-	Deletion of exons 46 - 48 (heterozygote)
45	5	30	44	BMD	M	-	+	-	+	18	+	37	+	37	c.10498_10499delAG (p.3498_3499del)
46	2	17	27	BMD	M	-	-	-	-	-	-	-	+	21	c.10129A>T (p.K3377X)
47	3	40	40	BMD	M	-	-	-	-	-	-	-	ND	ND	Deletion of exons 45 – 47
48	1	6	10	DMD	M	-	-	-	+	10	-	-	-	-	c.8249delA (p.D2750fs)
49	30	34	36	BMD	M	-	-	-	-	-	-	-	-	-	Duplications of exons 63 - 79
50	3	22	23	BMD	M	-	-	-	-	-	-	-	ND	ND	c.434G>C (p.Arg145Pro)
51	7	19	24	DMD	M	-	+	-	+	11	+	18	+	19	Deletion of exon 51
52	4	30	30	BMD	M	-	-	-	-	-	-	-	-	-	Deletion of exons 45 - 48
53	12	12	43	BMD	M	-	+	-	+	17	+	38	+	38	Deletion of exons 45 - 54
54	7	22	37	DMD	M	+	+	-	+	8	+	22	+	22	c.433C>T (p.Arg145Ter)
55	4	11	39	DMD	M	-	+	-	+	6	+	25	+	34	Deletion of exons 44
56	3	9	23	DMD	M	+	+	-	+	9	+	17	+	16	c.10108C>T (p.Arg3370Ter)
57	5	9	19	DMD	M	-	+	-	+	7	+	18	-	-	c.9568C>T (p.Arg3190Ter)
58	7	28	40	DMD	M	+	+	-	+	12	+	37	-	-	Deletion of exons 3 - 7
59	20	34	34	BMD	M	+	-	-	-	-	-	-	-	-	Deletion of exons 45 - 47
60	5	26	29	BMD	M	+	-	-	-	-	-	-	+	28	Deletion of exons 45 - 48
60-1	10	17	19	BMD	M	+	-	-	-	-	-	-	-	-	Deletion of exons 45 - 48
61	6	28	31	BMD	M	-	-	-	+	16	+	31	-	-	**c.8219_8228delACCTCCAA GG(p.Asp2740ValfsTer21)**
62	5	8	27	DMD	M	+	+	-	+	11	+	27	+	27	Deletion of exon 45
63	10	28	28	BMD	M	-	-	-	-	-	-	-	ND	ND	Deletion of exons 45 - 55
64	15	19	19	BMD	M	+	-	-	-	-	-	-	ND	ND	c.9225-647A>G
65	5	6	6	Symptomatic carrier	F	-	-	-	-	-	-	-	ND	ND	Deletion of exons 10-17 (heterozygous)
66	8	31	31	Symptomatic carrier	F	-	-	-	-	-	-	-	ND	ND	**c.491delCinsTT (p.Ser164PhrfsTer2)** (heterozygous)
67	11	30	30	BMD	M	-	-	-	-	-	-	-	ND	ND	Deletion of exons 45 - 55
68	5	5	26	DMD	M	-	+	-	+	9	+	18	-	-	c.433C>T (p.Arg145Ter)
69	6	22	22	BMD	M	-	-	-	-	-	-	-	ND	ND	Deletion of exons 45 - 47
70	23	29	54	BMD	M	+	-	-	-	-	-	-	ND	ND	**c.3374C>A (p.Ser1125Ter)**
71	1	10	20	DMD	M	-	+	13	+	11	+	-	-	-	Duplication of exon 2
72	7	10	34	IMD	M	-	-	-	+	14	+	26	-	-	c.10454delT (p.Leu3485ArgfsTer11)
73	6	30	39	DMD	M	-	+	-	+	12	+	22	-	-	c.2111delC (p.Pro704HisfsTer25)
74	6	32	36	DMD	M	-	+	-	+	12	+	26	+	36	c.649+1G>T
75	5	16	19	DMD	M	+	+	16	+	10	+	26	-	-	c.10223+1G>A

76	3	7	19	IMD	M	-	+	-	+	14	-	-	-	-	c.133C>T(p.Gln45Ter)
77	10	16	40	BMD	M	-	+	-	+	32	+	-	+	34	c.3603+2dupT
78	4	7	21	DMD	M	-	+	11	+	10	+	10	ND	ND	Deletion of exons 46 - 48
79	2	14	33	DMD	M	-	+	-	+	12	+	18	+	31	Deletion of exon 12
80	4	10	31	DMD	M	+	+	-	+	12	+	5	+	18	**c.4171A>T(p.Lys1391Ter)**
81	6	15	23	DMD	M	+	+	12	+	8	+	25	+	19	c.10223+1G>A
82	4	18	18	DMD	M	-	+	-	+	9	+	18	+	18	Deletion of exons 44 - 48
83	6	8	28	DMD	M	+	+	-	+	12	+	20	+	26	Duplication of exons 5 - 6
84	2	4	18	DMD	M	-	+	-	+	10	+	18	ND	ND	c.4471_4472delAA
85	6	8	28	IMD	M	-	+	-	+	14	+	28	+	28	Duplication of exons 3 - 18
86	2	26	33	BMD	M	+	-	-	-	-	-	-	+	26	Duplication of exons 14 - 29
87	3	10	24	DMD	M	+	+	-	+	6	+	16	-	-	Deletion of exons 45 - 50
88	5	23	23	DMD	M	-	+	-	+	12	+	23	-	-	Deletion of exons 46 - 47
89	5	7	26	DMD	M	+	+	-	+	11	+	26	-	-	Deletion of exons 46 - 48
90	8	20	20	BMD	M	-	-	-	-	-	-		ND	ND	Deletion of exons 45 - 49
91	8	7	26	DMD	M	-	+	-	+	11	+	23	+	23	Deletion of exons 46 - 48
92	10	15	27	DMD	M	-	-	-	+	12	+	27	ND	ND	Deletion of exon 50
93	3	20	40	IMD	M	+	+	-	+	14	+	34	-	-	Deletion of exons 45 - 52
94		36	45	Symptomatic carrier	F	-	-	-	+	45	-	-	ND	ND	Deletion of exons 48 - 52 (heterozygote)
95	4	8	24	IMD	M	+	+	18	+	13	+	20	+	20	Deletion of exon 45
96	5	13	33	IMD	M	+	+	-	+	13	+	27	+	27	Duplication of exons 20 - 55
97	2	25	42	IMD	M	-	+	-	+	13	+	25	-	-	Deletion of exons 12 - 13
98	12	26	35	BMD	M	+	+	-	+	27	-	-	-	-	Deletion of exons 3 - 7
99	7	6	18	IMD	M	-	+	12	+	13	+	19	+	18	Deletion of exons 8 - 16
100	2	7	25	DMD	M	+	+	-	+	9	+	22	-	-	Deletion of exons 46 - 51
101	8	17	25	DMD	M	+	+	-	+	12	+	18	+	42	c.4729C>T (p.Arg1577Ter)
102	3	5	31	DMD	M	-	+	17	+	12	+	21	+	28	Deletion of exons 48 - 50
103	2	9	13	DMD	M	+	+	-	+	10	-	-	-	-	Deletion of exons 45 - 52
104	4	8	26	DMD	M	-	+	14	+	10	+	17	-	-	Deletion of exons 10 - 44
105	13	33	45	BMD	M	+	+	-	+	19	+	30	+	33	Deletion of exons 3 - 7
106	3	16	28	DMD	M	+	+	-	+	12	+	17	+	25	Deletion of exon 44
107	2	7	16	DMD	M	-	+	-	+	12	+	12	ND	ND	Duplication of exons 8 - 15
108	2	3	24	DMD	M	+	+	16	+	10	+	18	-	-	Deletion of exons 46 - 51
109	2	7	25	DMD	M	-	+	-	+	12	+	22	-	-	Duplication of exon 2
110	5	12	36	DMD	M	-	+	-	+	12	+	20	+	33	Deletion of exon 45
111	7	20	26	DMD	M	-	+	-	+	11	+	21	-	-	Deletion of exons 45 - 52
112	6	23	37	DMD	M	-	+	-	+	10	+	24	-	-	Deletion of exons 48 - 52
113	5	25	34	DMD	M	-	+	-	+	10	+	25	+	25	Deletion of exons 45 - 54
114	6	19	19	DMD	M	-	+	-	+	12	+	20	+	19	Deletion of exons 49 - 50
115	7	16	24	DMD	M	-	+	-	+	10	+	22	-	-	Deletion of exons 44 - 60
116	7	16	18	DMD	M	-	+	-	+	10	+	16	+	16	Deletion of exon 43
117	9	30	34	IMD	M	-	+	-	+	13	+	33	+	32	Deletion of exons 3 - 6
118	4	9	17	DMD	M	-	+	12	+	10	-	-	-	-	Deletion of exons 49 - 50
119	7	13	27	DMD	M	-	+	15	+	10	+	19	-	-	Deletion of exons 45 - 50
120	13	21	26	BMD	M	-	-	-	-	-	-	-	+	22	Deletion of exons 45 - 53
121	6	9	9	DMD	M	-	-	-	+	9	-	-	ND	ND	Deletion of exon 55
122	5	4	10	DMD	M	-	-	-	+	11	-	-	-	-	Deletion of exons 1 - 13

123	5	29	29	DMD	M	-	+	-	+	11	+	30	+	29	Deletion of exons 45 - 52
124	6	12	19	DMD	M	-	+	12	+	10	+	19	-	-	Duplication of exons 30 - 49
125	2	10	30	IMD	M	-	+	-	+	13	+	30	-	-	Deletion of exons 2 - 16
126	2	14	15	DMD	M	+	+	15	+	8	-	8	+	14	Deletion of exons 46 - 47
127	3	7	7	UD	M	-	+	-	-	-	-	-	ND	ND	Deletion of exons 49 - 50
128	5	24	24	DMD	M	-	+	-	+	12	+	24	-	-	Deletion of exons 42 - 43
129	7	8	19	BMD	M	+	-	-	-	-	-	-	-	-	Deletion of exons 45 - 55
129-1	3	2	19	BMD	M	+	-	-	-	-	-	-	ND	ND	Deletion of exons 45 - 55
130	9	27	32	DMD	M	-	+	-	+	11	+	28	-	-	Deletion of exons 46 - 47
131	7	8	26	DMD	M	-	+	13	+	11	+	15	+	17	c.8800G>T (p.Glu2934Ter)<br>(Hemizygote)
132	8	10	32	DMD	M	-	+	-	+	12	+	23	+	23	Deletion of exon 51
133	3	9	9	DMD	M	-	+	-	+	10	-	-	-	-	Deletion of exons 50 - 52
134	9	16	16	BMD	M	-	-	-	-	-	-	-	ND	ND	Deletion of exons 45 - 47
135	20	33	38	BMD	M	+	-	-	-	-	+	-	+	34	Duplication of exons 3 - 13
135-1	16	17	33	BMD	M	+	-	-	-	-	-	-	ND	ND	Duplication of exons 3 - 13
136	5	12	25	IMD	M	-	+	-	+	15	-	-	ND	ND	Duplication of exons 19 - 50
137	6	17	17	DMD	M	-	+	17	+	10	+	17	+	17	Deletion of exon 51
138	5	31	43	DMD	M	-	-	-	+	11	+	31	-	-	Deletion of exons 4 - 7
139	3	5	18	DMD	M	+	+	13	+	10	+	16	-	-	Duplications of exons 2 - 7<br>and 45 – 51
140	6	6	17	DMD	M	-	+	-	+	9	+	-	+	17	Deletion of exons 10 - 21
141	8	23	39	DMD	M	-	+	-	+	11	+	24	+	23	Deletion of exons 31 - 43
142	4	5	7	UD	M	-	-	-	-	-	-	-	ND	ND	Deletion of exons 45 - 54
143	8	10	10	UD	M	-	-	-	-	-	-	-	ND	ND	Deletion of exons 44 - 50
144	6	23	36	IMD	M	-	+	-	+	13	+	23	+	23	Duplication of exon 12
145	1	15	21	DMD	M	-	+	-	+	11	+	19	+	17	Deletion of exons 70 - 71
146	5	8	29	BMD	M	-	-	-	-	-	-	-	-	-	Deletion of exons 45 - 48
147	8	16	31	IMD	M	-	+	-	+	14	+	26	-	-	Duplication of exon 44
147-1	9	16	37	IMD	M	+	+	-	1	14	1	26	-	-	Duplication of exon 44
148	11	38	39	BMD	M	-	-	-	+	17	+	38	+	38	Deletion of exons 7 - 17
149	1	13	34	DMD	M	-	+	22	+	9	+	23	-	-	Deletion of exon 45
150	3	4	10	UD	M	-	-	-	-	-	-	-	-	-	Deletion of exons 49 - 50
151	7	9	15	DMD	M	-	+	-	+	11	-	-	ND	ND	Deletion of exon 51
152	3	11	17	DMD	M	-	+	-	+	9	+	-	+	16	Deletion of exon 52
153	5	3	29	DMD	M	+	+	-	+	10	+	20	+	23	Duplications of exons 5 - 37<br>and 50 – 59
154	1	0	1	UD	M	+	-	-	-	-	-	-	ND	ND	Duplication of exons 61 - 62
155	3	2	6	UD	M	-	-	-	-	-	-	-	ND	ND	Deletion of exons 3 - 44
156	6	6	6	UD	M	-	-	-	-	-	-	-	-	-	Deletion of exons 45 - 47
157	6	6	18	DMD	M	-	+	14	+	9	+	-	-	-	Deletion of exons 46 - 47
158	7	6	9	UD	M	-	-	-	-	-	-	-	ND	ND	Deletion of exons 3 - 7
159	5	14	22	DMD	M	-	+	-	+	10	+	15	-	-	Deletion of exon 50
160	4	6	8	UD	M	-	-	-	-	-	-	-	ND	ND	Deletion of exons 45 - 50
161	3	10	23	BMD	M	-	+	-	-	-	-	-	-	-	Deletion of exons 45 - 53
162	8	11	22	DMD	M	-	+	-	+	11	+	20	+	26	Deletion of exons 46 - 52
163	4	6	12	DMD	M	+	+	-	+	8	-	-	ND	ND	Deletion of exons 10 - 11
164	15	34	40	BMD	M	-	-	-	-	-	-	-	-	-	Deletion of exons 45 - 49

165	6	8	24	DMD	M	-	+	13	+	9	+	16	+	23	Deletion of exon 22
166	4	14	20	DMD	M	+	+	14	+	10	+	15	-	-	Deletion of exons 49 - 51
167	8	27	27	BMD	M	-	-	-	-	-	-	-	ND	ND	Deletion of exons 45 - 47
168	5	7	29	DMD	M	+	-	-	+	12	+	20	-	-	Duplication of exons 3 - 7
169	1	22	28	DMD	M	-	+	-	+	9	+	22	-	-	Deletion of exon 44
170	5	24	38	DMD	M	+	+	-	+	10	+	25	+	26	Duplication of exons 12 - 19
171	8	7	8	UD	M	-	-	-	-	-	-	-	ND	ND	Deletion of exons 45 - 47
172	22	27	35	BMD	M	-	+	-	-	-	+	-	-	-	Deletion of exons 45 - 47
173	3	3	15	DMD	M	-	+	15	+	11	+	-	ND	ND	Duplication of exons 2 - 9
174	6	7	31	DMD	M	+	+	-	+	11	+	20	+	21	Deletion of exons 46 - 50
175	3	14	35	DMD	M	+	+	-	+	11	+	17	+	27	Deletion of exons 52 - 54
176	4	10	22	DMD	M	-	+	14	+	11	+	13	+	15	Deletion of exons 46 - 52
177	6	7	9	UD	M	-	-	-	-	-	-	-	ND	ND	Duplication of exons 14 - 45
178	9	23	41	IMD	M	-	+	-	+	13	+	23	+	28	Deletion of exons 20 - 37
179	5	5	10	DMD	M	-	-	-	+	10	-	-	ND	ND	Deletion of exons 47 - 52
180	11	11	12	BMD	M	-	+	-	-	-	-	-	-	-	Deletion of exons 45 - 47
181	6	10	17	DMD	M	+	+	17	+	11	+	16	-	-	Duplication of exon 53
181-1	10	12	12	DMD	M	+	+	-	1	11	1	-	ND	ND	Duplication of exon 53
182	5	7	11	DMD	M	-	+	-	+	10	-	-	ND	ND	Deletion of exon 61
183	4	4	30	IMD	M	-	+	-	+	13	+	22	-	-	Deletion of exons 8 - 28
184	13	13	18	BMD	M	-	-	-	-	-	-	-	ND	ND	Deletion of exons 45 - 47
185	4	4	24	DMD	M	+	+	-	+	10	+	22	+	19	Deletion of exons 49 - 50
186	1	9	22	DMD	M	-	+	14	+	9	+	14	+	15	Deletion of exons 3 - 11
187	7	15	15	DMD	M	+	+	-	+	9	-	-	-	-	Duplications of exons 52 - 53 and 56 – 61
188	5	23	33	IMD	M	-	+	-	+	14	+	27	-	-	Deletion of exons 42 - 43
189	5	28	33	DMD	M	+	-	-	+	10	-	-	+	28	Deletion of exons 3 - 7
190	6	15	30	DMD	M	-	+	-	+	9	+	22	+	21	Duplication of exons 53 - 54
191	8	7	15	BMD	M	+	-	-	-	-	-	-	ND	ND	Duplication of exons 3 - 37
192	3	24	32	DMD	M	-	+	-	+	11	+	24	+	35	Deletion of exons 48 - 50
193	2	7	18	DMD	M	+	+	18	+	10	+	-	+	18	Deletion of exons 48 - 52
194	4	12	18	IMD	M	+	+	-	+	14	-	-	ND	ND	Deletion of exons 46 - 52
195	6	14	17	BMD	M	+	-	-	-	-	-	-	ND	ND	Deletion of exon 45 - 49
196	7	7	7	UD	M	-	-	-	-	-	-	-	ND	ND	Deletion of exon 44
197	23	33	39	BMD	M	-	-	-	-	-	-	-	-	-	Deletion of exons 45 - 47
198	5	8	24	DMD	M	-	+	-	+	11	+	22	+	22	Deletion of exons 44 - 48
199	6	6	28	IMD	M	-	+	-	+	13	+	28	+	28	Deletion of exons 49 - 50
200	4	5	24	BMD	M	-	-	-	-	-	-	-	ND	ND	Deletion of exons 45 - 47
201	3	13	13	DMD	M	-	+	-	+	8	-	-	-	-	Duplications of exons 52-53 and 56-61
202	2	3	16	DMD	M	-	+	-	+	9	+	15	-	-	Duplication of exon 8
203	8	9	23	IMD	M	-	+	-	+	15	+	-	-	-	Deletion of exon 51
204	8	6	26	DMD	M	-	+	-	+	8	+	9	+	18	Deletion of exon 51
205	3	6	20	DMD	M	-	+	-	+	11	+	17	-	-	Deletion of exon 51
206	16	16	28	BMD	M	-	+	-	+	22	-	-	-	-	Deletion of exons 45 - 49
207	13	22	48	BMD	M	+	-	-	-	-	-	-	-	-	Deletion of exons 45 - 48
207-1	19	19	25	BMD	M	+	-	-	-	-	-	-	ND	ND	Deletion of exons 45 - 48
208	8	8	12	DMD	M	+	-	-	+	12	-	-	ND	ND	Deletion of exon 45

209	3	1	3	UD	M	+	-	-	-	-	-	-	ND	ND	Deletion of exons 60 - 79
210	3	7	22	DMD	M	-	+	-	1	12	1	18	+	19	Deletion of exons 43 - 45
211	2	7	15	DMD	M	-	+	18	1	11	1	14	+	23	Duplication of exon 55
212	3	21	29	DMD	M	-	+	-	1	8	1	22	+	21	Deletion of exons 18 - 44
213	6	13	23	DMD	M	-	+	-	1	10	1	21	-	-	Deletion of promoter region - exon 51
214	7	15	22	BMD	M	-	+	-	1	18	1	22	-	-	Deletion of exons 45 - 46
215	3	5	5	UD	M	-	-	-	-	-	-	-	ND	ND	Duplication of exon 2
216	5	5	5	UD	M	-	-	-	-	-	-	-	ND	ND	Deletion of exons 45 - 55
217	6	5	6	UD	M	-	-	-	-	-	-	-	ND	ND	Deletion of exons 45 - 47
218	20	31	49	BMD	M	+	+	-	1	37	1	47	+	49	Duplication of exons 2 - 7

Hx, history; Dx, diagnosis; FH, family history; DCMP, dilated cardiomyopathy; DMD, Duchenne muscular dystrophy; BMD, Becker muscular dystrophy; IMD, intermediate phenotype muscular dystrophy; UD, undetermined phenotype; ND, echocardiography was not done. Bold text indicates novel variants.
